# ﻿Genomic investigation of benthic invertebrates from the Clarion-Clipperton fields of polymetallic nodules

**DOI:** 10.3897/zookeys.1231.135347

**Published:** 2025-03-10

**Authors:** Romain Gastineau, Kamila Mianowicz, Przemysław Dąbek, Christian Otis, Valcana Stoyanova, Artur Krawcewicz, Tomasz Abramowski

**Affiliations:** 1 Institute of Marine and Environmental Sciences, University of Szczecin, ul. Mickiewicza 16a, Szczecin, 70-383, Poland University of Szczecin Szczecin Poland; 2 Interoceanmetal Joint Organization, ul. Cyryla i Metodego 9-9A, Szczecin, 71-541, Poland Interoceanmetal Joint Organization Szczecin Poland; 3 Plateforme d'Analyse Génomique, Institut de biologie intégrative et des systèmes, Université Laval, Québec, QC, Canada Université Laval Québec Canada; 4 Maritime University of Szczecin, ul. Wały Chrobrego 1-2, Szczecin, 70-500, Poland Maritime University of Szczecin Szczecin Poland

**Keywords:** Brachiopoda, Bryozoa, genome-skimming, Holothuroidea, introns, Mitochondrial genomes, Ophiuroidea, Polychaeta, Porifera, ribosomal RNA genes

## Abstract

The abyssal plains of the Clarion-Clipperton Zone (CCZ) are famous for their fields of polymetallic nodules, which are inhabited by benthic invertebrates. Ten specimens from the Interoceanmetal Joint Organisation (IOM) licence area in the CCZ were collected in 2014 and submitted to a short-read genome skimming sequencing. In total, mitochondrial genomes and nuclear ribosomal genes were retrieved for nine different organisms belonging to Ophiuroidea, Holothuroidea, Polychaeta, Bryozoa, Porifera, and Brachiopoda (assigned to these phyla immediately upon retrieval from the seafloor). As many of these samples were partial and physically deteriorated following their seven-year storage in IOM’s collections, their morphology-based taxonomic identification could rarely be performed at the lowest possible level (species or genus) prior to preparing the samples for molecular or genomic investigations. Therefore, it was not possible to apply the reverse identification scheme recommended for such investigations. However, several of these specimens represent poorly studied groups for which few molecular references are available as of now. In two cases, the presence of introns in the mitochondrial genome questions the practicability of using the *cox1* gene for further routine molecular barcoding of these organisms. These results might be useful in future DNA primers design, molecular barcoding, and phylogeny or population genetic studies when more samples are obtained.

## ﻿Introduction

Located in the Pacific Ocean, the Clarion-Clipperton Zone (CCZ) spans 4.5 million km^2^ between Hawaii and Mexico. The abyssal plain of this area has recently become a focus of attention due to the massive presence of polymetallic nodule deposits on its floor, which hold potential for exploitation. Far from being a lifeless environment, the floor of the CCZ is inhabited by benthic fauna (Rabone et al. 2023), mostly composed of invertebrates (e.g., Amon et al. 2016; Christodoulou et al. 2019) and large benthic foraminifera (e.g., Stachowska-Kamiǹska et al. 2022; Gooday and Wawrzyniak-Wydrowska 2023). Although the scale of environmental impacts of nodule exploitation activity (deep-sea mining) is yet to be fully understood, the retrieval of the resource from the seafloor is likely to affect benthic fauna, especially the sessile species which live attached to the nodules.

Environmental considerations have led the International Seabed Authority (ISA) to issue a certain number of recommendations in order to assess benthic biodiversity in the licence areas as part of the baseline studies. Environmental baseline studies are conducted by ISA contractors holding exploration licences with the aim to describe this benthic environment. The findings of the studies will constitute a basis (the baseline) against which exploitation impacts will be assessed, and are to be incorporated into an informed decision-making process by both ISA (e.g., while preparing standards and guidelines and defining environmental thresholds) and contractors (e.g., while preparing environmental management and monitoring plans).

Barcoding and more generally genetic studies are some of the tools used to identify and/or support taxonomic identification of fauna – in particular, key and representative species that could be used as indicator for assessing impacts – collected during exploration activities (ISBA/25/LTC/6/Rev.3 2023). This taxonomic work is of primary importance in species cataloguing and biodiversity assessment. In addition, molecular studies help to unravel the ecological functions and connectivity of species or assemblages (Danovaro et al. 2017). Although reverse taxonomy is advocated (ISBA/25/LTC/6/Rev.3 2023), this approach is not always possible when revisiting legacy data and specimen collections, as in the case in this article. Fully aware of the limitations resulting from the lack of proper morphology-based taxonomic identification of specimens retrieved from the IOM licence area in 2014, we nevertheless decided to try a genome-skimming approach on these samples.

IOM conducts exploration activities in the area located in the eastern part of the CCZ under the contract signed with ISA in 2001. Recently, IOM has developed its own protocol for the molecular study of the CCZ benthic fauna (Gastineau et al. 2023). This protocol emphasizes the use of the genome-skimming approach based on short-read sequencing whenever possible, with the aim of obtaining the largest amount of data possible within a single sequencing. In the best-case scenario, the outcome could be a complete cluster of nuclear rRNA genes and/or a complete mitochondrial genome. This is exemplified by the aforementioned article on *Abyssoprimnoagemina* Cairns, 2015 (Gastineau et al. 2023). This deep-sea coral was collected during the IOM cruise to the CCZ in 2014, together with the specimens described in the present article. All the specimens were documented by macrophotography immediately upon retrieval but were not taxonomically identified to the species level at that time, except in a few cases, including *A.gemina*. The samples had been stored in ethanol 96% for seven years before the molecular and genomic protocol was implemented. This, in addition to the fact that several of these samples were partial, resulted in many cases in their poor physical conservation, which made it impossible to perform proper morphology-based taxonomic identification. Moreover, it has to be stressed that in the case of the CCZ fauna, there are still many species that remain undescribed (Rabone et al. 2023). Even if references exist for some of these taxa, it might still be difficult to identify them at the species level, considering the scarcity of taxonomic expertise.

When applying a basic molecular barcoding protocol to the 2014 samples to amplify the *18S* and *cox1* genes, we faced several challenges. As much as we could generally obtain positive results for the *18S* gene, we mostly failed to amplify the *cox1* gene, regardless of the phyla. This was likely a consequence of the DNA primers not being sufficiently specific rather than insufficient quality or quantity of the DNA extracted. Indeed, in several cases, the amount of DNA retrieved qualified the samples for a next-generation type of sequencing as previously performed on *A.gemina* (Gastineau et al. 2023).

In the current article, we present the results of a genome-skimming strategy applied to ten samples from the CCZ that represent two species of Ophiuroidea, one Holothuroidea, one Polychaeta, two Bryozoa, two Porifera, and one Brachiopoda. Of these samples, only the Ophiuroidea specimens could be identified at the species level (morphological identification confirmed by molecular results). It has not been possible to identify or describe the other samples at the species level so far, but our findings still hold some potential for the scientific community involved in the exploration of the CCZ. Some of these samples may represent poorly studied phyla for which few molecular references are available. Some others have mitogenomes with complex features that could not be resolved by the usual PCR and Sanger sequencing protocol, which in some cases render the amplification of their *cox1* gene impossible.

## ﻿Materials and methods

### ﻿Exploration, sampling, photographic documentation, and storage

**Figure 1.**
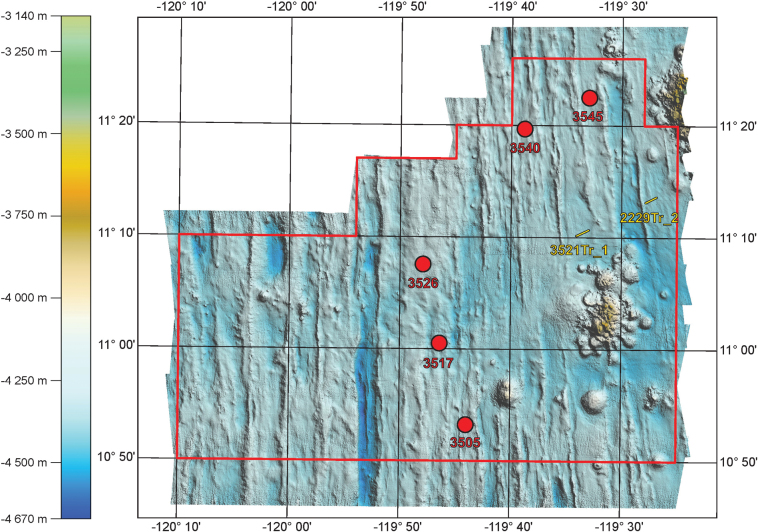
Location of the sampling stations in the IOM claim area.

All the specimens sequenced during this study were collected during the 2014 IOM cruise. Two different sampling methods were used: point (box coring) and linear (trawling) sampling. The sample names, coordinates and depths of the sampling stations are given in Table 1, while their location is shown in Fig. 1. Polymetallic nodules and their sessile or associated fauna were retrieved from the seafloor and photographed onboard the research vessel using a Nikon D700 camera equipped with an AF-S MICRO Nikkor 105 mm 1:2.8G ED lens. All the samples were then stored in 2.0 mL Eppendorf tube filled with 96% ethanol and stored at 4 °C (the cold chain protocol was applied). They were all assigned accession numbers in the collection of IOM.

**Table 1.** Name, coordinates and depth of the sampling stations.

Station	Point / line (sampling method)	Coordinates	Depth
3505	point (box core)	10°53,2380'N, 119°43,9970'W	4332 m
3517	point (box core)	11°00,5100'N, 119°46,4130'W	4325 m
3526	point (box core)	11°07,5510'N, 119°47,9380'W	4241 m
3540	point (box core)	11°19,6800'N, 119°38,8230'W	4249 m
3545	point (box core)	11°22,4940'N, 119°32,9930'W	4285 m
2229Tr_2	line (trawl)	From 11°13,1495'N, 119°27,8542'W to 11°13,5805'N, 119°26,9792'W	4307–4310 m
3521Tr_1	line (trawl)	From 11°10,1623'N, 119°34,0946'W to 11°10,6813'N, 119°32,9836'W	4265–4291 m

### ﻿DNA extraction, sequencing and bioinformatic analyses

DNA was extracted using the DNeasy Blood & Tissue extraction kit from Qiagen following the instructions of the manufacturer. All DNA samples were sent to the Beijing Genomics Institute in Shenzhen, China to be sequenced on a DNBSEQ platform for an average number of 60 million 100-bp paired-end reads per sample. The reads were assembled using SPAdes 3.15.5 (Bankevich et al. 2012) and a k-mer of 85. The sequences of interest (mitogenomes and rRNA nuclear clusters) were extracted from the contigs file by customized blastn queries (Camacho et al. 2009) using similar sequences from GenBank as reference. The boundaries of the rRNA nuclear genes were localized with Rfam 14 (Kalvari et al. 2021). Mitochondrial genomes were annotated using MITOS 2.1.9 (Donath et al. 2019) and uploaded to the OGDRAW server to obtain their maps (Lohse et al. 2013). For the sake of clarity, all the maps are presented in a circular form, including those of the mitogenomes with no redundant endings. Megablast queries of the complete *18S* and *cox1* genes of each specimen were performed on the NCBI blast server. The specimens were given scientific names according to the World Register of Marine Species (WoRMS). When needed, maximum likelihood molecular phylogenies were performed using IQ-TREE 2.2.0 (Minh et al. 2020) with 1000 ultrafast bootstrap replicates following selection of the best model of evolution with ModelTest-NG (Darriba et al. 2020).

### ﻿Data resources

All the clean sequencing reads were deposited on SRA with accession number PRJNA1130051. All the mitochondrial genomes and the rRNA genes (complete clusters or partial) are available on GenBank with the accession numbers given in the Results section.

## ﻿Results

### ﻿Specimen IOM_2014_13: unidentified Demospongiae

Fig. 2.

**Figure 2.**
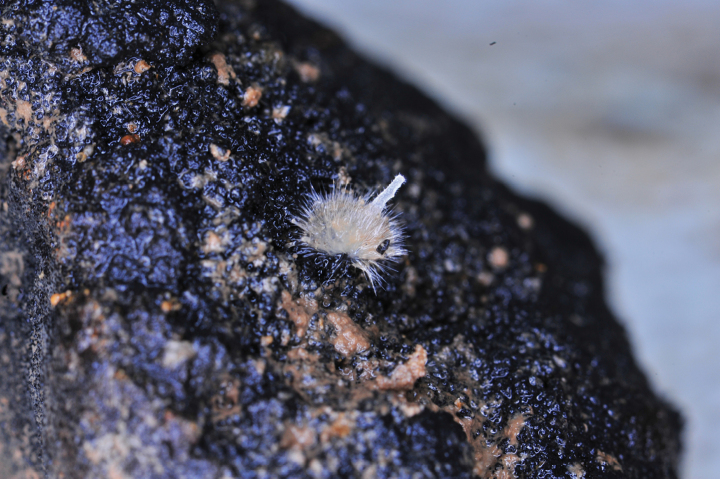
Specimen IOM_2014_13 on a polymetallic nodule immediately after sampling (unscaled).

Station ID: 3503.

Biosample: SAMN42180853.

#### ﻿Cluster of nuclear rRNA genes

The cluster is complete with a total length of 6,022 bp (GenBank: PP968935). The best *18S* megablast result is *Polymastiapachymastia* voucher UCMPWC932 (GenBank: EF654528) (Kober and Nichols 2007), E-value 0.0, identity 99.93% for a length of 1,660 bp. However, it should be noted that a 100% identity was found with the shorter reference PP848924 from *Spinularia* sp. voucher RC1570, for a length of 782 bp (Lim et al. 2024). After trimming the *28S* part to its variable D1/D2 region, megablast query returned a 100% identity with the 782 bp sequence of *Spinularia* sp. voucher RC1570 (GenBank: PP848924).

#### ﻿Mitochondrial genome

**Figure 3.**
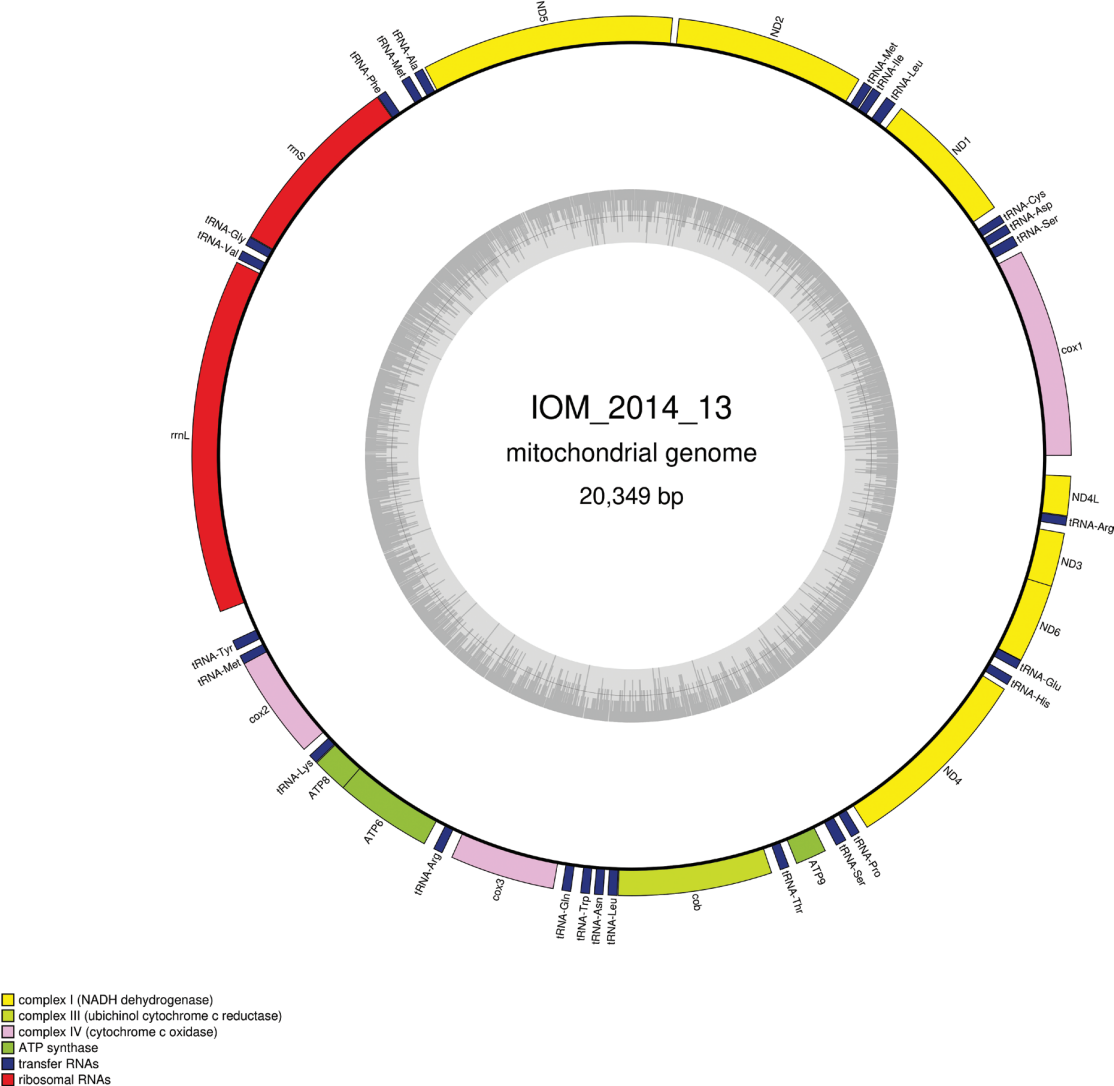
Map of the mitochondrial genome of specimen IOM_2014_13, with the type of genes indicated by colour boxes and the GC content indicated by the grey circle.

The mitogenome was found complete with redundant endings (GenBank: PP971517). It is 20,349 bp long and encodes 14 protein coding genes, two rRNAs and 25 tRNAs (Fig. 3, Table 2). All genes are encoded on the same strand. The nucleotide composition is A (30.89%), T (36.92%), C (12.36%) and G (19.82%). The genome is colinear with those of *Polymastialittoralis* Stephens, 1915 (GenBank: KJ129611) (del Cerro et al. 2016). The *cox1* megablast query returned a 100% identity with *Radiellasarsii* Ridley & Dendy, 1886 specimen voucher ZMBN:98039 (GenBank: HG423721) (Plotkin et al. 2017) for a length of 658 bp, and also with *Spinularia* sp. voucher RC1570 (GenBank: PP851905) for a length of 656 bp. The currently accepted name of *R.sarsi* is *Spinulariasarsii* Ridley & Dendy, 1886 (de Voogd et al. 2024).

**Table 2.** Characteristics of the genes encoded by the mitogenome of the unidentified Demospongiae IOM_2014_13.

Gene	Strand	Location	Size (bp)	Start codon	Stop codon	Anticodon
*cox1*	+	1–1563	1563	ATG	TAG	
*tRNA-Ser*	+	1613–1696	84			TGA
*tRNA-Asp*	+	1722–1793	72			GTC
*tRNA-Cys*	+	1810–1876	67			GCA
*ND1*	+	1941–2951	1011	ATG	TAG	
*tRNA-Leu*	+	3003–3086	84			TAA
*tRNA-Ile*	+	3135–3207	73			GAT
*tRNA-Met*	+	3216–3286	71			CAT
*ND2*	+	3324–4739	1416	ATG	TAA	
*ND5*	+	4782–6674	1893	ATG	TAG	
*tRNA-Ala*	+	6690–6762	73			TGC
*tRNA-Met*	+	6801–6872	72			CAT
*tRNA-Phe*	+	7009–7081	73			GAA
*rrnS*	+	7082–8486	1405			
*tRNA-Gly*	+	8487–8558	72			TCC
*tRNA-Val*	+	8602–8674	73			TAC
*rrnL*	+	8696–11349	2654			
*tRNA-Tyr*	+	11578–11657	80			GTA
*tRNA-Met*	+	11701–11772	72			CAT
*cox2*	+	11725–12543	819	ATG	TAA	
*tRNA-Lys*	+	12600–12672	73			TTT
*ATP8*	+	12674–12946	273	ATG	TAG	
*ATP6*	+	12940–13674	735	ATG	TAA	
*tRNA-Arg*	+	13748–13821	74			TCT
*cox3*	+	13895–14683	789	ATG	TAG	
*tRNA-Gln*	+	14743–14814	72			TTG
*tRNA-Trp*	+	14883–14953	71			TCA
*tRNA-Asn*	+	14984–15054	71			GTT
*tRNA-Leu*	+	15085–15158	74			TAG
*cob*	+	15160–16314	1155	ATG	TAA	
*tRNA-Thr*	+	16377–16450	74			TGT
*ATP9*	+	16506–16742	237	ATG	TAA	
*tRNA-Ser*	+	16828–16913	86			GCT
*tRNA-Pro*	+	16955–17027	73			TGG
*ND4*	+	17090–18541	1452	ATG	TAA	
*tRNA-His*	+	18576–18648	73			GTG
*tRNA-Glu*	+	18712–18783	72			TTC
*ND6*	+	18781–19380	600	ATG	TAA	
*ND3*	+	19371–19778	408	ATG	TAA	
*tRNA-Arg*	+	19832–19902	71			TCG
*ND4L*	+	19903–20202	300	ATG	TAG	

### ﻿Specimen IOM_2014_15: unidentified Bryozoa

Fig. 4.

**Figure 4.**
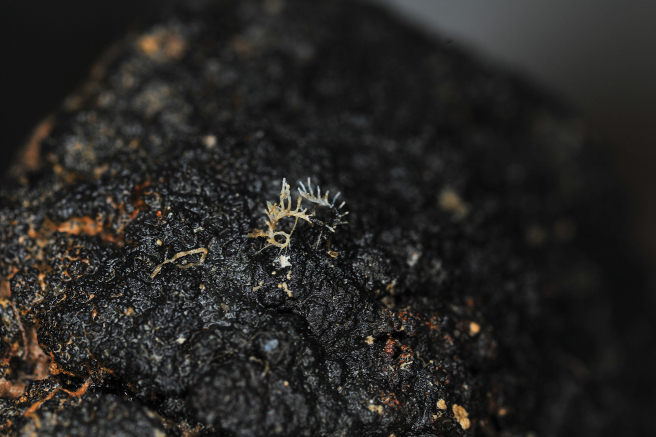
Specimen IOM_2014_15 on a polymetallic nodule immediately after sampling (unscaled).

Station ID: 3517.

Biosample: SAMN42180854.

#### ﻿Cluster of nuclear rRNA genes

The cluster is complete with a total length of 6,768 bp (GenBank: PP968936). The best *18S* megablast result is *Tubuliporalobifera* Hastings, 1963 (GenBank: JN680934) (Waeschenbach et al. 2012), E-value 0.0, identity 96.96% for a length of 1,812 bp.

#### ﻿Mitochondrial genome

**Figure 5.**
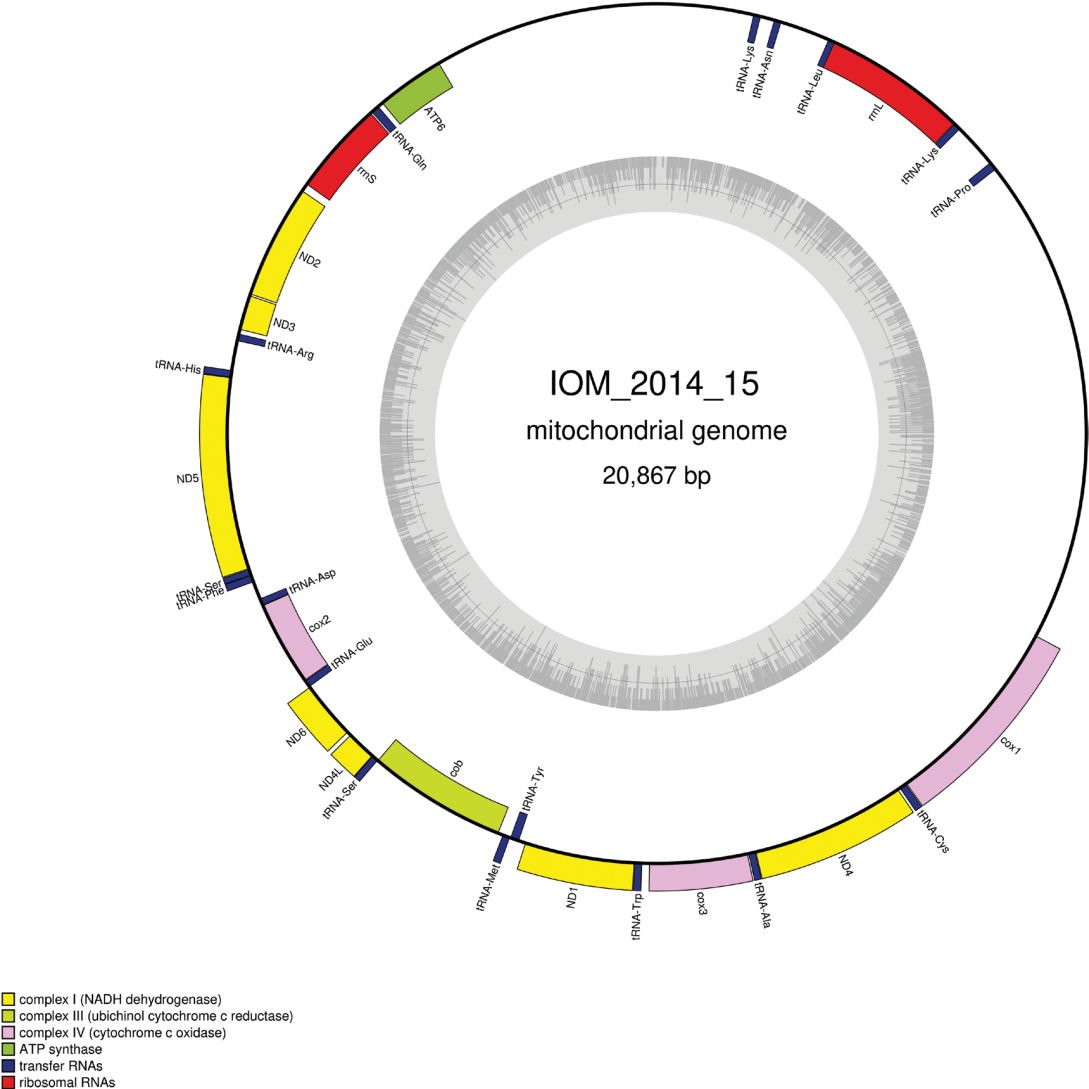
Map of the mitochondrial genome of specimen IOM_2014_15, with the type of genes indicated by colour boxes and the GC content indicated by the grey circle.

The mitogenome has no redundant endings but seems to contain all conserved genes (GenBank: PP990757). For easier reading, it is represented as circular. It is 20,867 bp long and encodes 12 protein coding genes, two rRNAs and 19 tRNAs, encoded on both strands (Fig. 5, Table 3). The nucleotide composition is A (38.54%), T (39.94%), C (11.53%) and G (9.98%). No *ATP8* could be found. There are two large non-coding regions between *tRNA-Lys* and *ATP6* and between *tRNA-Pro* and *cox1*. The best *cox1* megablast result is *Tubuliporaflabellaris* (O. Fabricius, 1780) (GenBank: NC_015646) (Sun et al. 2011), E-value 0.0, identity 80.12%. This reference sequence is also a mitochondrial genome that is not colinear with those of specimen 2014_15.

**Table 3.** Characteristics of the genes encoded by the mitogenome of the unidentified Bryozoa IOM_2014_15.

Gene	Strand	Location	Size (bp)	Start codon	Stop codon	Anticodon
*tRNA-Pro*	-	2198..2267	70			TGG
*tRNA-Lys*	-	2615..2674	60			TTT
*rrnL*	-	2676..3810	1135			
*tRNA-Leu*	-	3796..3852	57			TAA
*tRNA-Asn*	-	4240..4298	59			GTT
*tRNA-Lys*	-	4404..4463	60			TTT
*ATP6*	-	6981..7520	540	ATA	TAA	
*tRNA-Gln*	-	7558..7627	70			TTG
*rrnS*	-	7636..8380	745			
*ND2*	-	8438..9337	900	ATG	TAA	
*ND3*	-	9347..9622	276	ATG	TAA	
*tRNA-Arg*	-	9654..9712	59			TCG
*tRNA-His*	+	9942..9998	57			GTG
*ND5*	+	9998..11497	1500	ATG	TAG	
*tRNA-Ser*	+	11499..11548	53			TCT
*tRNA-Phe*	+	11548..11604	57			GAA
*tRNA-Asp*	-	11742..11798	57			GTC
*cox2*	-	11797..12465	669	ATG	TAA	
*tRNA-Glu*	-	12466..12525	60			TTC
*ND6*	+	12527..12985	459	ATG	TAA	
*ND4L*	+	13013..13249	237	ATG	TAA	
*tRNA-Ser*	+	13248..13303	56			TGA
*cob*	-	13302..14396	1095	ATG	TAA	
*tRNA-Met*	+	14432..14492	61			CAT
*tRNA-Tyr*	-	14495..14565	71			GTA
*ND1*	+	14617..15477	861	ATG	TAA	
*tRNA-Trp*	+	15478..15537	60			TCA
*cox3*	+	15591..16358	768	ATG	TAA	
*tRNA-Ala*	+	16364..16419	56			TGC
*ND4*	+	16420..17631	1212	ATG	TAA	
*tRNA-Cys*	+	17646..17704	59			GCA
*cox1*	+	17707..19242	1536	ATG	TAA	

### ﻿Specimen IOM_2014_17: unidentified Polychaeta

Fig. 6.

**Figure 6.**
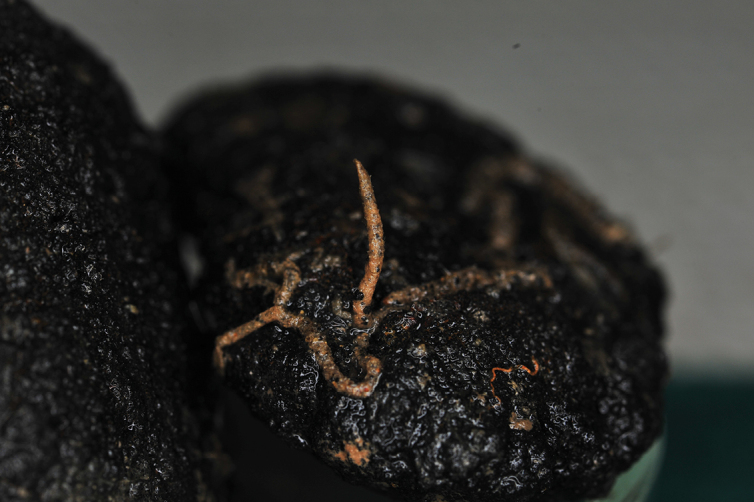
Specimen IOM_2014_17 on a polymetallic nodule immediately after sampling (unscaled).

Station ID: 3517.

Biosample: SAMN42180855.

#### ﻿Cluster of nuclear rRNA genes

The cluster is complete with a total length of 6,365 bp (GenBank: PP970526). The best *18S* megablast result is *Nicomachelumbricalis* Fabricius, 1780 isolate SPM24 (GenBank: MG975479) (Eilertsen et al. 2018), E-value 0.0, identity 99.87% for a length of 1,552 bp.

#### ﻿Mitochondrial genome

**Figure 7.**
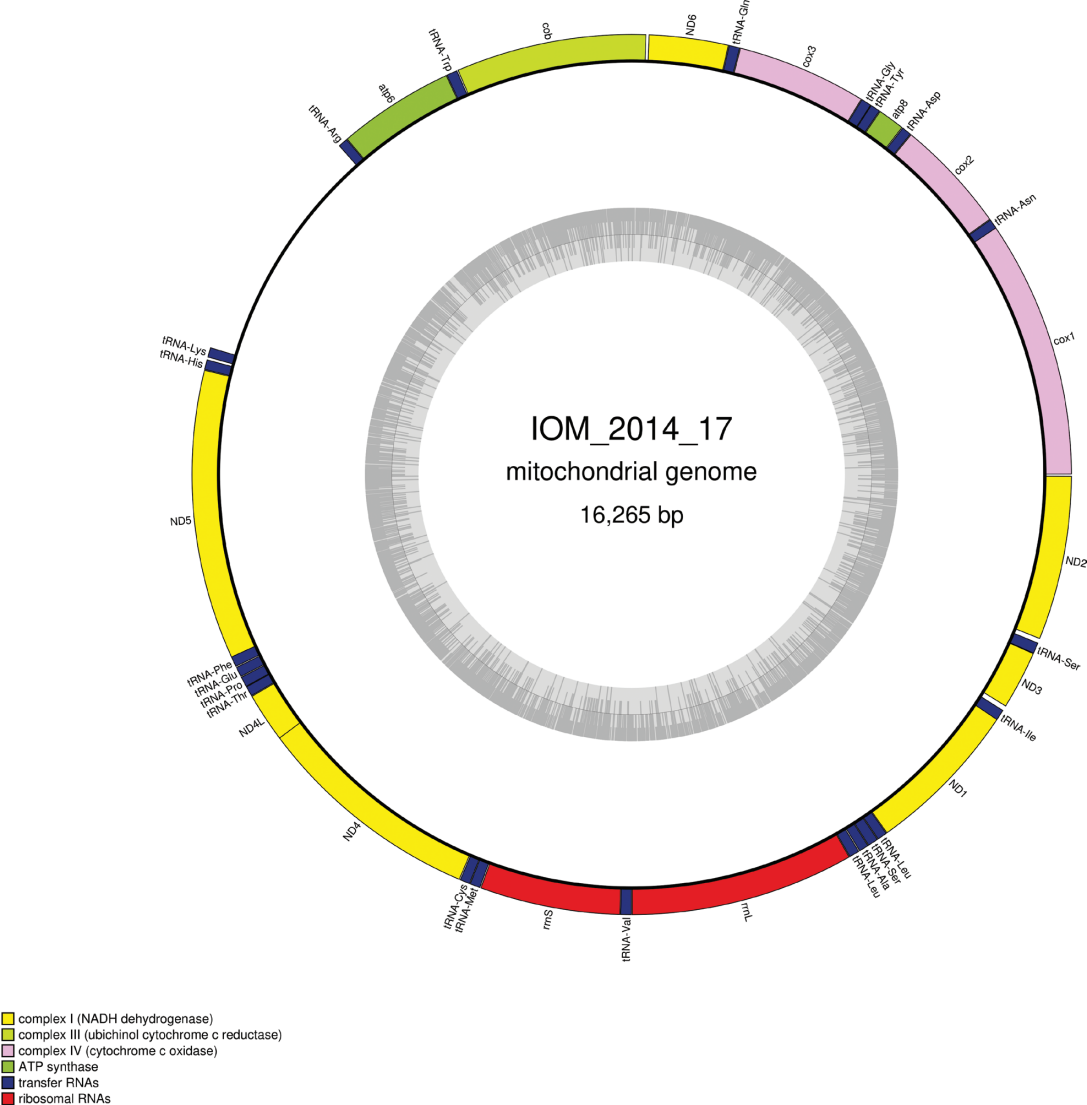
Map of the mitochondrial genome of specimen IOM_2014_17, with the type of genes indicated by colour boxes and the GC content indicated by the grey circle.

The mitogenome is complete with redundant endings (GenBank: PP990759). It is 16,265 bp long and encodes 13 protein coding genes, two rRNAs and 22 tRNAs, all on the same strand (Fig. 7, Table 4). The nucleotide composition is A (30.70%), T (32.66%), C (22.98%) and G (13.66%). The best *cox1* megablast result is Nicomachecf.benthaliana NHM_058 (GenBank: OQ271976), which is also found in the CCZ (Stewart et al. 2023), with E-value 0.0, identity 99.28% for a length of 554 bp.

**Table 4.** Characteristics of the genes encoded by the mitogenome of the unidentified Polychaeta IOM_2014_17. T(AA) in the stop codon column indicates a premature termination with the addition of 3’ A residues to the mRNA.

Gene	Strand	Location	Size (bp)	Start codon	Stop codon	Anticodon
*cox1*	+	1–1537	1537	ATG	T(AA)	
*tRNA-Asn*	+	1538–1599	62			GTT
*cox2*	+	1600–2284	685	ATG	TAA	
*tRNA-Asp*	+	2285–2348	64			GTC
*atp8*	+	2350–2512	163	ATG	TAA	
*tRNA-Tyr*	+	2513–2577	65			GTA
*tRNA-Gly*	+	2577–2641	65			TCC
*cox3*	+	2642–3421	780	ATG	TAG	
*tRNA-Gln*	+	3422–3489	68			TTG
*ND6*	+	3490–3966	477	ATG	TAA	
*cob*	+	3980–5119	1140	ATG	TAA	
*tRNA-Trp*	+	5123–5190	68			TCA
*ATP6*	+	5191–5890	700	ATG	T(AA)	
*tRNA-Arg*	+	5891–5951	61			TCG
*tRNA-Lys*	+	7374–7435	62			TTT
*tRNA-His*	+	7448–7510	63			GTG
*ND5*	+	7511–9236	1726	ATG	T(AA)	
*tRNA-Phe*	+	9237–9302	66			GAA
*tRNA-Glu*	+	9305–9368	64			TTC
*tRNA-Pro*	+	9370–9434	65			TGG
*tRNA-Thr*	+	9434–9497	64			TGT
*ND4L*	+	9498–9797	300	ATG	TAA	
*ND4*	+	9791–11158	1368	ATG	TAA	
*tRNA-Cys*	+	11161–11223	63			GCA
*tRNA-Met*	+	11224–11288	65			CAT
*rrnS*	+	11291–12131	841			
*tRNA-Val*	+	12132–12199	68			TAC
*rrnL*	+	12200–13540	1341			
*tRNA-Leu*	+	13541–13605	65			TAG
*tRNA-Ala*	+	13608–13669	62			TGC
*tRNA-Ser*	+	13670–13736	67			TGA
*tRNA-Leu*	+	13737–13799	63			TAA
*ND1*	+	13800–14733	934	ATG	T(AA)	
*tRNA-Ile*	+	14734–14802	69			GAT
*ND3*	+	14829–15182	354	ATG	TAA	
*tRNA-Ser*	+	15181–15249	69			TCT
*ND2*	+	15274–16257	984	ATG	TAA	

### ﻿Specimen IOM_2014_38: *Silaxdaleus* Lyman, 1879, Ophiuroidea

Fig. 8.

**Figure 8.**
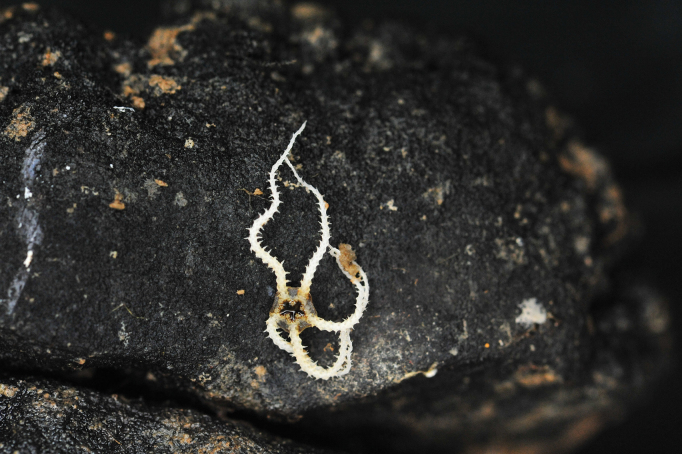
Specimen IOM_2014_38 identified as *Silaxdaleus* on a polymetallic nodule immediately after sampling (unscaled).

Station ID: 3526.

Biosample: SAMN42180856.

#### ﻿Cluster of nuclear rRNA genes

The cluster is complete with a total length of 6,940 bp (GenBank: PP970577). The best *18S* megablast result is Amphiopluscf.daleus NHM_447 (KU519529) (Glover et al. 2016), E-value 0.0, identity 100.00% for a length of 1,676 bp. *Amphioplusdaleus* is a synonym of *Silaxdaleus* (Stöhr et al. 2024). Manual alignment of the *28S* gene with the two partial sequences of Amphiopluscf.daleus (GenBank: MN170903 and MN170901, 993 bp and 973 bp long, respectively) (Christodoulou et al. 2019) showed a complete identity with MN170903 and three polymorphisms at the very end of the 3’ part of MN170901.

#### ﻿Mitochondrial genome

**Figure 9.**
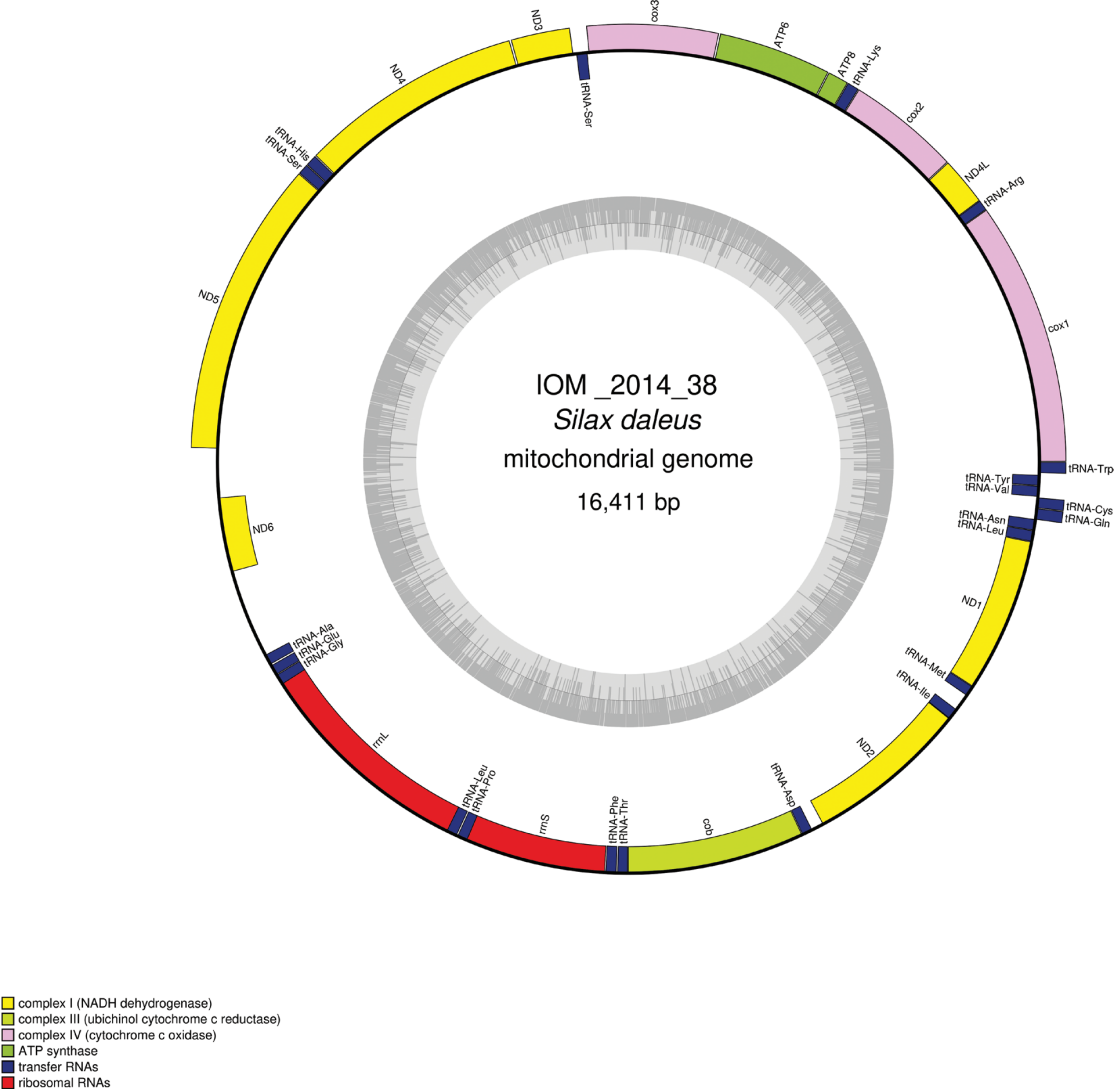
Map of the mitochondrial genome of *Silaxdaleus* specimen IOM_2014_38, with the type of genes indicated by colour boxes and the GC content indicated by the grey circle.

The mitogenome is complete with redundant endings (GenBank: PP977505). It is 16,411 bp long and encodes 13 protein coding genes, two rRNAs and 22 tRNAs encoded on both strands (Fig. 9, Table 5). The nucleotide composition is A (34.82%), T (30.25%), C (21.54%) and G (13.39%). The best *cox1* megablast result is *Amphioplusdaleus* voucher SO242_1_181_D4 (GenBank: MT160448) (Christodoulou et al. 2020), E-value 0.0, identity 100% for a length of 658 bp. The mitogenome is colinear with those of *Amphiurasinicola* (GenBank: NC_045938) (Lee et al. 2019), another representative of the Amphiuridae family whose accepted name is currently Amphiura (Fellaria) sinicola Matsumoto, 1941. Both mitogenomes share identical features, including the the premature ending of the protein-coding genes *cob* and *ND1* by the presence of a tRNA.

**Table 5.** Characteristics of the genes encoded by the mitogenome of Silaxdaleus specimen IOM_2014_38. T(AA) in the stop codon column indicates a premature termination with the addition of 3’ A residues to the mRNA.

Gene	Strand	Location	Size (bp)	Start codon	Stop codon	Anticodon
*cox1*	+	1–1602	1602	ATG	TAA	
*tRNA-Arg*	+	1603–1669	67			TCG
*ND4L*	+	1670–1966	297	ATG	TAA	
*cox2*	+	1969–2655	687	ATG	TAA	
*tRNA-Lys*	+	2657–2730	74			CTT
*ATP8*	+	2732–2860	129	ATG	TAA	
*ATP6*	+	2864–3550	687	ATG	TAA	
*cox3*	+	3558–4355	798	ATG	TAG	
*tRNA-Ser*	-	4364–4435	72			TGA
*ND3*	+	4454–4813	360	ATG	TAA	
*ND4*	+	4822–6183	1362	ATG	TAA	
*tRNA-His*	+	6187–6258	72			GTG
*tRNA-Ser*	+	6260–6326	67			GCT
*ND5*	+	6328–8121	1794	ATG	TAA	
*ND6*	-	8433–8912	480	ATG	TAA	
*tRNA-Ala*	-	9481–9550	70			TGC
*tRNA-Glu*	-	9558–9625	68			TTC
*tRNA-Gly*	-	9627–9697	71			TCC
*rrnL*	-	9673–11123	1451			
*tRNA-Leu*	-	11112–11182	71			TAG
*tRNA-Pro*	-	11188–11255	68			TGG
*rrnS*	-	11250–12158	909			
*tRNA-Phe*	-	12162–12232	71			GAA
*tRNA-Thr*	-	12238–12304	67			TGT
*cob*	-	12305–13448	1144	GTG	T(AA)	
*tRNA-Asp*	-	13450–13519	70			GTC
*ND2*	-	13595–14650	1056	GTG	TAG	
*tRNA-Ile*	-	14650–14723	74			GAT
*tRNA-Met*	-	14832–14900	69			CAT
*ND1*	-	14901–15900	1000	GTG	T(AA)	
*tRNA-Leu*	-	15901–15972	72			TAA
*tRNA-Asn*	-	15973–16045	73			GTT
*tRNA-Gln*	+	16044–16115	72			TTG
*tRNA-Cys*	+	16117–16184	68			GCA
*tRNA-Val*	-	16189–16258	70			TAC
*tRNA-Tyr*	-	16260–16328	69			GTA
*tRNA-Trp*	+	16340–16410	71			TCA

### ﻿Specimen IOM_2014_51: Bryozoa

Fig. 10.

**Figure 10.**
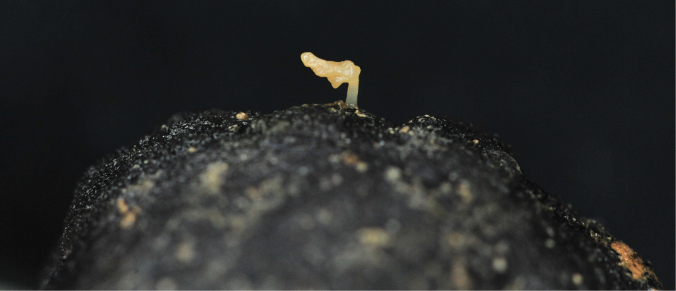
Specimen IOM_2014_51 on a polymetallic nodule immediately after sampling (unscaled).

Station ID: 3540.

Biosample: SAMN42180857.

#### ﻿Cluster of nuclear rRNA genes

The cluster is complete with a total length of 6,530 bp (GenBank: PP971152). The best *18S* megablast result is *Hornerafoliacea* MacGillivray, 1869 (GenBank: FJ409613) (Waeschenbach et al. 2009), E-value 0.0, identity 98.64% for a length of 1,810 bp.

#### ﻿Mitochondrial genome

**Figure 11.**
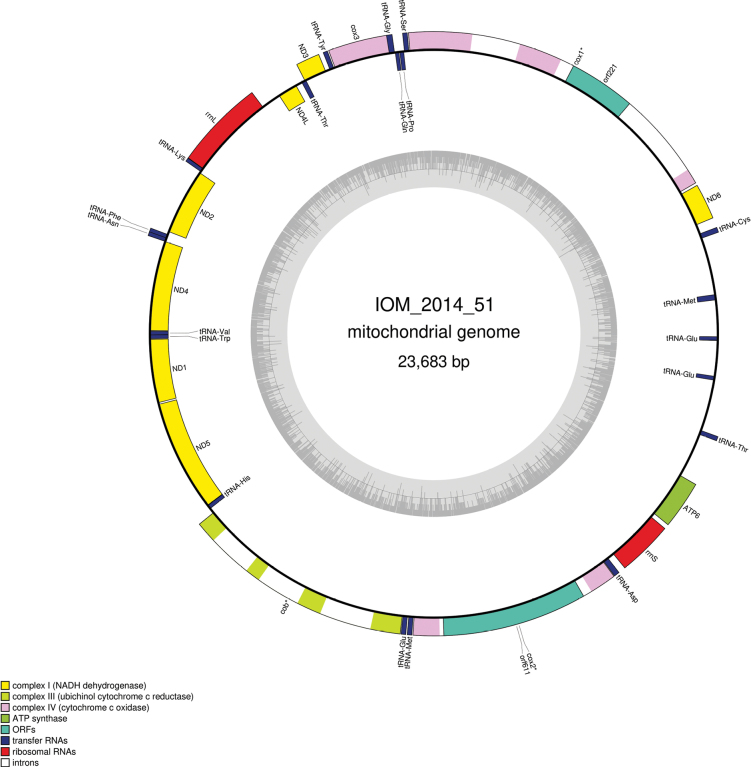
Map of the mitochondrial genome of specimen IOM_2014_51, with the type of genes indicated by colour boxes and the GC content indicated by the grey circle. Introns appear in white.

The mitogenome has no redundant endings but seems to contain all conserved genes (GenBank: PP990758). Having 23,683 bp, the mitogenome is long and rather complex. It contains 12 protein coding genes, 16 tRNAs and two rRNAs encoded on both strands (Fig. 11, Table 6). The nucleotide composition is A (36.65%), T (39.00%), C (9.61%) and G (14.74%). There are group II introns in *cox1* (two introns), *cox2* (one intron) and *cob* (three introns). The intron in *cox2* contains an open-reading frame coding for a putative reverse transcriptase. The intron in *cox1* also contains an open-reading frame, but only the maturase domain seems conserved and complete. No *ATP8* could be found. The best megablast result for the coding sequence (CDS) of *cox1* was not relevant and did not relate to Bryozoa, which was also the case when blasting the full mitogenome.

**Table 6.** Characteristics of the genes encoded by the mitogenome the unidentified Bryozoa IOM_2014_51. The size of the intron-containing genes is indicated with and without the introns.

Gene	Strand	Location	Size (bp)	Start codon	Stop codon	Anticodon
*tRNA-Met*	-	450–527	78			CAT
*tRNA-Cys*	+	1291–1360	70			GCA
*ND6*	+	1482–1940	459	ATG	TAA	
*cox1*	+	1969–6242 (2 introns)	4274 (full) 1518 (CDS)	ATG	TAA	
*tRNA-Ser*	+	6251–6315	65			TCT
*tRNA-Pro*	-	6320–6377	58			TGG
*tRNA-Gln*	-	6384–6438	55			TTG
*tRNA-Gly*	+	6442–6518	77			TCC
*cox3*	+	6497–7264	768	ATG	TAA	
*tRNA-Tyr*	+	7276–7337	62			GTA
*tRNA-Arg*	+	7339–7404	66			TCG
*ND3*	+	7392–7700	309	ATG	TAG	
*tRNA-Thr*	-	7680–7746	67			TGT
*ND4L*	-	7828–8094	267	ATG	TAA	
*rrnL*	+	8353–9501	1149			
*tRNA-Lys*	+	9500–9552	53			TTT
*ND2*	-	9567–10457	891	ATG	TAA	
*tRNA-Phe*	+	10491–10547	57			GAA
*tRNA-Asn*	+	10540–10595	56			GTT
*ND4*	-	10591–11799	1209	ATG	TAA	
*tRNA-Val*	-	11802–11856	55			TAC
*tRNA-Trp*	-	11851–11906	56			TCA
*ND1*	-	11910–12770	861	ATG	TAA	
*ND5*	-	12788–14290	1503	ATG	TAA	
*tRNA-His*	-	14290–14346	57			GTG
*cob*	+	14410–17344 (3 introns)	2935 (full) 1113 (CDS)	ATG	TAA	
*tRNA-Glu*	+	17349–17413	65			TTC
*tRNA-Met*	+	17426–17489	64			CAT
*cox2*	+	17497–20172 (2 introns)	2676 (full) 666 (CDS)	ATG	TAA	
*tRNA-Asp*	+	20175–20250	76			GTC
*rrnS*	+	20336–21044	709			
*ATP6*	+	21099–21716	618	ATG	TAA	
*tRNA-Thr*	+	22313–22374	62			GGT
*tRNA-Glu*	-	23064–23121	58			TTC
*tRNA-Glu*	-	23592–23649	58			TTC

### ﻿Specimens IOM_2014_54 and 2014_58: *Ophiosphalmaglabrum* (Lütken & Mortensen, 1899)

Figs 12, 13.

**Figure 12.**
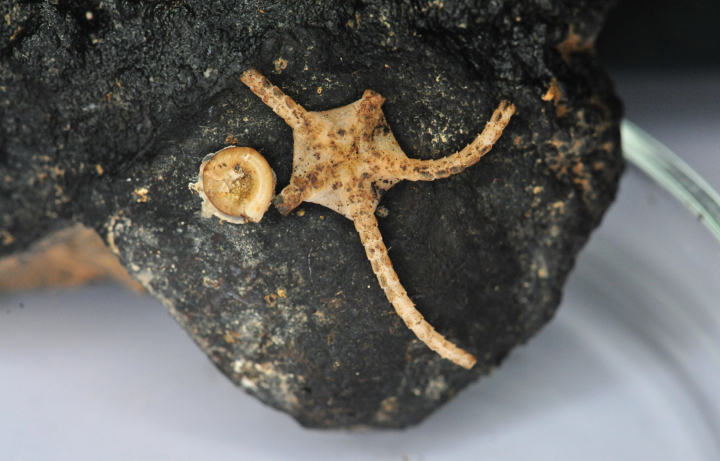
Specimen IOM_2014_54 on a polymetallic nodule immediately after sampling (unscaled).

**Figure 13.**
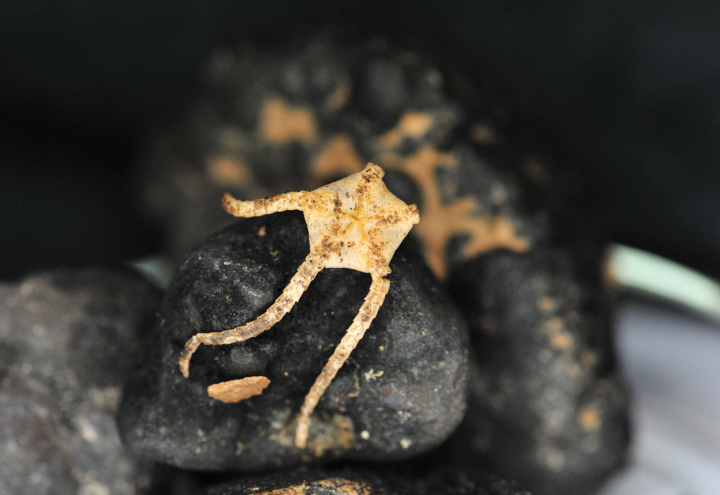
Specimen IOM_2014_58 on a polymetallic nodule immediately after sampling (unscaled).

Station ID IOM_2014_54: 2229Tr_2; Station ID IOM_2014_58: 3521Tr_1.

Biosample IOM_2014_54: SAMN46122295; Biosample IOM_2014_58: SAMN46122296.

#### ﻿Cluster of nuclear rRNA genes

We failed at assembling the cluster of rRNA and could only retrieve the *18S* gene (GenBank: PP960805 and PP968762 for specimens IOM_2014_54 and IOM_2014_58 respectively). Both are 1816 bp long and are 100% identical with each other. The best *18S* megablast result is Ophiomusiumcf.glabrum NHM_329 (GenBank: KU519536) from Glover et al. (2016), E-value 0.0, identity 99.82% for a length of 1669 bp. *Ophiomusiumglabrum* is a non-accepted synonym of *Ophiosphalmaglabrum*.

#### ﻿Mitochondrial genome

**Figure 14.**
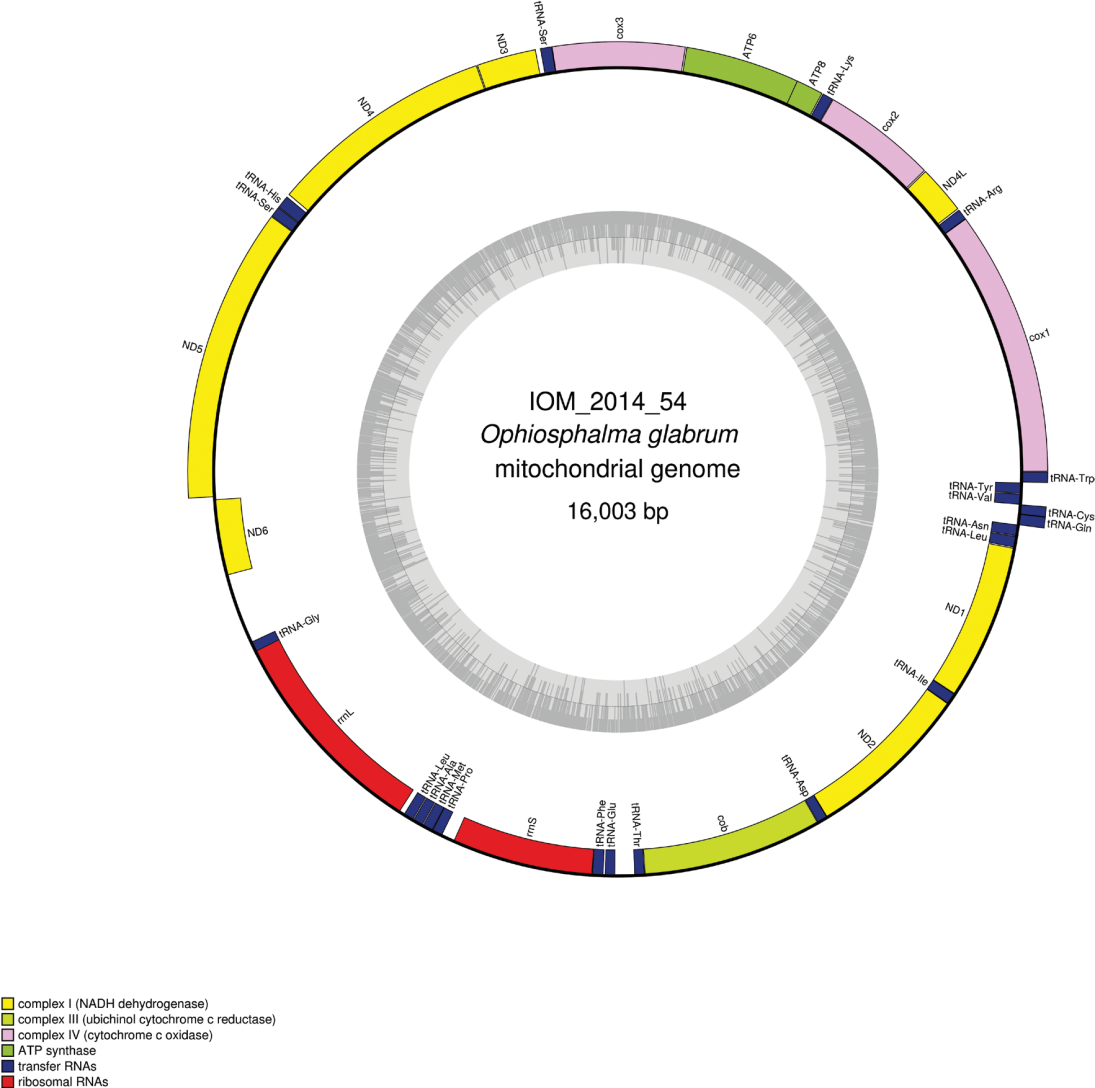
Map of the mitochondrial genome of *Ophiosphalmaglabrum* specimen IOM_2014_54, with the type of genes indicated by colour boxes and the GC content indicated by the grey circle.

**Figure 15.**
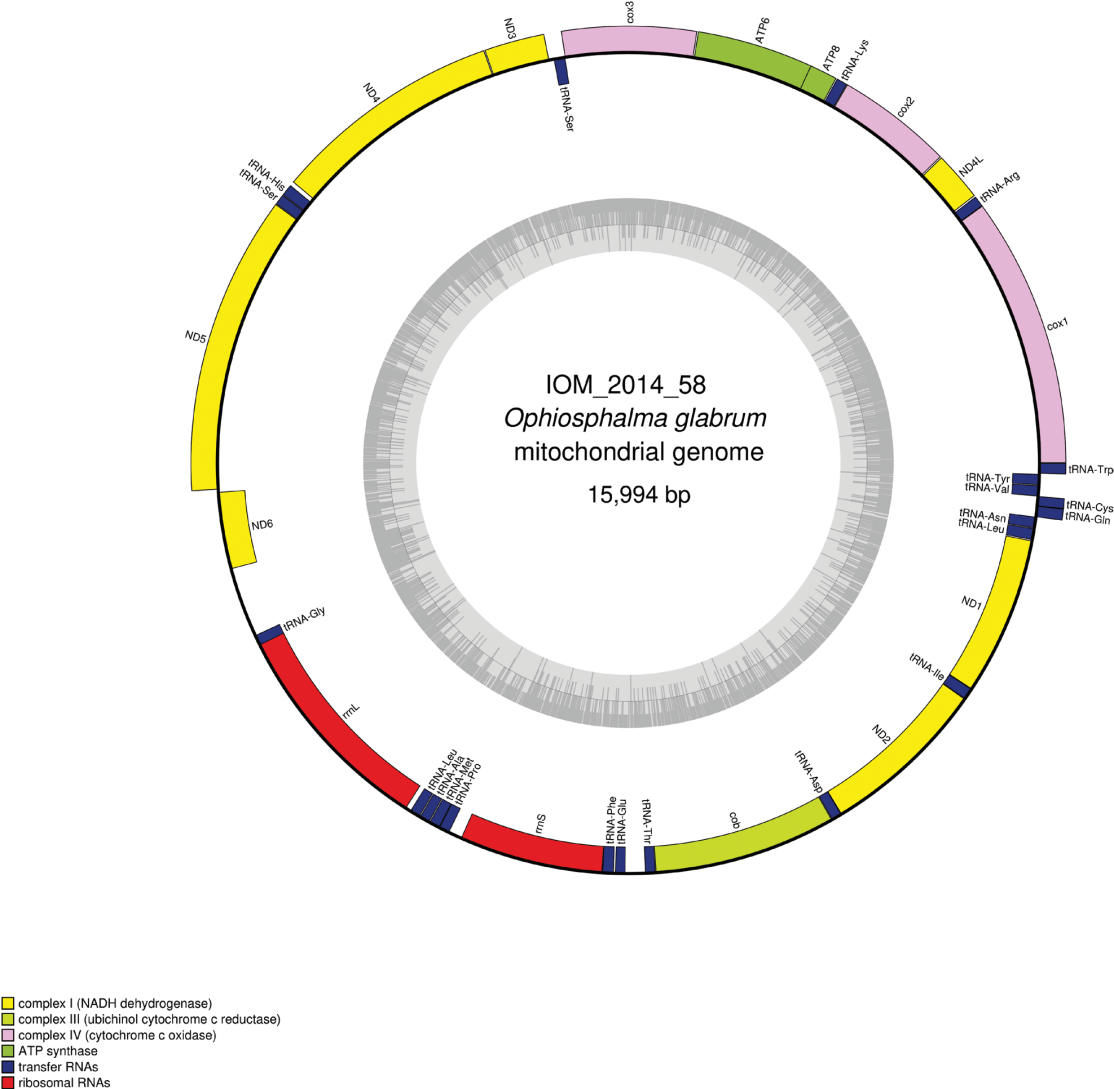
Map of the mitochondrial genome of *Ophiosphalmaglabrum* specimen IOM_2014_58, with the type of genes indicated by colour boxes and the GC content indicated by the grey circle.

The mitogenomes were found complete with redundant endings. They are 16,003 bp long for specimen 2014_54 (GenBank: PP977506) and 15,994 bp long for 2014_58 (GenBank: PP977508). The mitogenomes encode for 13 protein coding genes, two rRNA and 22 tRNA, encoded on both strands (Figs 14, 15, Table 7). The nucleotide composition is A (35.09%/35.10%), T (33.44%/33.46%), C (19.43%/19.43%) and G (12.05%/12.01%) for IOM_2014_54 and IOM_2014_58, respectively. Both *cox2* and *cob* have premature termination by the presence of a tRNA. The best megablast results for the *cox1* gene were *Ophiosphalmaglabrum* voucher DSB_3935 (GenBank: MW770847), E-value 0.0, identity 99.70% for a length of 658 bp for IOM_2014_54, and *Ophiosphalmaglabrum* voucher DSB_42 (GenBank: MW770844), E-value 0.0, identity 99.85% for a length of 653 bp for IOM_2014_58. In Table 8, a comparison for each gene is presented. The most conserved gene was *ND6* and the most polymorph *ATP8*. It is to note that an indel was found in the *rrnS* gene. Most of the protein encoded were impacted by these mutations, except for the *cox1*, *ND4L*, and *ND6* genes.

**Table 7.** Characteristics of the genes encoded by the mitogenomes of Ophiosphalmaglabrum specimens IOM_2014_54 and IOM_2014_58. T(AA) in the stop codon column indicates a premature termination with the addition of 3’ A residues to the mRNA. When there are discrepancies in the positions of the genes, they are indicated separately for IOM_2014_54 and IOM_2014_58, respectively.

Gene	Strand	Location	Size (bp)	Start codon	Stop codon	Anticodon
*cox1*	+	1–1602	1602	ATG	TAA	
*tRNA-Arg*	+	1601–1668	68			TCG
*ND4L*	+	1675–1971	297	ATG	TAA	
*cox2*	+	1976–2666	624	ATG	T(AA)	
*tRNA-Lys*	+	2667–2734	68			CTT
*ATP8*	+	2738–2905	168	ATG	TAA	
*ATP6*	+	2893–3591	699	ATG	TAA	
*cox3*	+	3596–4393	798	ATG	TAA	
*tRNA-Ser*	-	4392–4463	72			TGA
*ND3*	+	4490–4849/4492–4851	360	ATG	TAA	
*ND4*	+	4852–6219/4854–6221	1368	ATG	TAA	
*tRNA-His*	+	6243–6314/6244–6315	72			GTG
*tRNA-Ser*	+	6317–6379/6316–6382	63			GCT
*ND5*	+	6382–8160/6383–8161	1779	ATG	TAA	
*ND6*	-	8176–8643/8177–8662	468	ATG	TAA	
*tRNA-Gly*	-	9108–9176/9099–9167	69			TCC
*rrnL*	-	9166–10549/9157–10540	1384			
*tRNA-Leu*	-	10580–10647/10571–10638	68			TAG
*tRNA-Ala*	-	10650–10716/10641–10707	67			TGC
*tRNA-Met*	-	10719–10787/10710–10778	69			CAT
*tRNA-Pro*	-	10787–10855/10778–10846	69			TGG
*rrnS*	-	10937–11841/10928–11833	905/906			
*tRNA-Phe*	-	11841–11912/11833–11904	72			GAA
*tRNA-Glu*	-	11921–11986/11913–11977	66			TTC
*tRNA-Thr*	-	12108–12173/12099–12164	66			TGT
*cob*	-	12174–13323/12165–13314	1150	ATG	T(AA)	
*tRNA-Asp*	-	13324–13391/13315–13382	68			GTC
*ND2*	-	13390–14445/13381–14436	1056	ATG	TAA	
*tRNA-Ile*	-	14445–14518/14436–14509	74			GAT
*ND1*	-	14518–15519/14509–15510	1002	ATG	TAA	
*tRNA-Leu*	-	15523–15595/15514–15586	73			TAA
*tRNA-Asn*	-	15597–15668/15588–15659	72			GTT
*tRNA-Gln*	+	15667–15736/15658–15727	70			TTG
*tRNA-Cys*	+	15735–15797/15726–15788	63			GCA
*tRNA-Val*	-	15796–15865/15787–15856	70			TAC
*tRNA-Tyr*	-	15867–15935/15858–15926	69			GTA
*tRNA-Trp*	+	15937–16003/15928–15994	67			TCA

**Table 8.** Number of single nucleotide polymorphisms (SNPs) and percentage of identity between the protein-coding and rRNA genes of specimens Ophiosphalmaglabrum 2014_54 and 2014_58.

Gene	SNPs/total length	Identity (%)
*ATP6*	9/705	98.72
*ATP8*	5/168	97.02
*cob*	12/1150	98.96
*cox1*	12/1602	99.25
*cox2*	10/691	98.55
*cox3*	9/798	98.75
*ND1*	17/1002	98.30
*ND2*	7/1056	99.34
*ND3*	4/360	98.89
*ND4*	16/1368	98.83
*ND4L*	4/297	98.65
*ND5*	16/1779	99.10
*ND6*	1/486	99.79
*rrnL*	5/1384	99.64
*rrnS*	4 SNPs + 1 indel/905–906	NA

### ﻿Specimen IOM_2014_55: unidentified Holothuroidea

Fig. 16.

**Figure 16.**
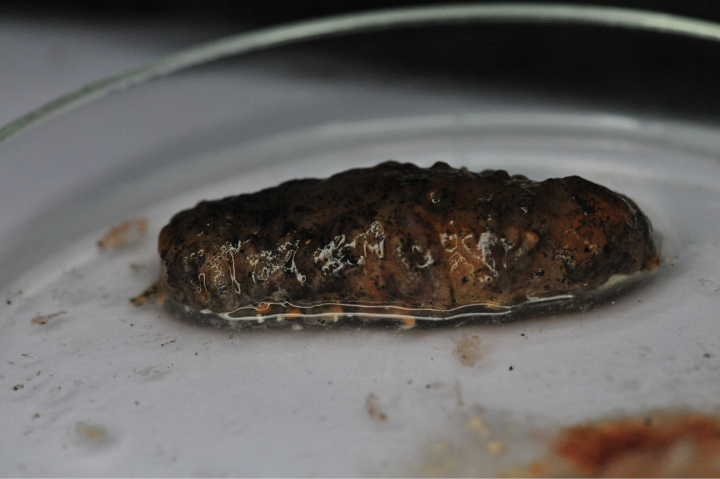
Specimen IOM_2014_55 after sampling, photo taken on a glass Petri dish after cleansing with water (unscaled).

Station ID: 2229Tr_2.

Biosample: SAMN42180858.

#### ﻿Cluster of nuclear rRNA genes

We failed at assembling a complete rRNA cluster. Instead, we extracted the *18S* gene, which is 1,876 bp long (GenBank: PP971153). The best *18S* megablast result is *Deimavalidum* (GenBank: KX856815), currently accepted name *Deimavalidumvalidum* Théel, 1879 (Miller et al. 2017), E-value 0.0, identity 99.34% for a length of 1,815 bp.

#### ﻿Mitochondrial genome

**Figure 17.**
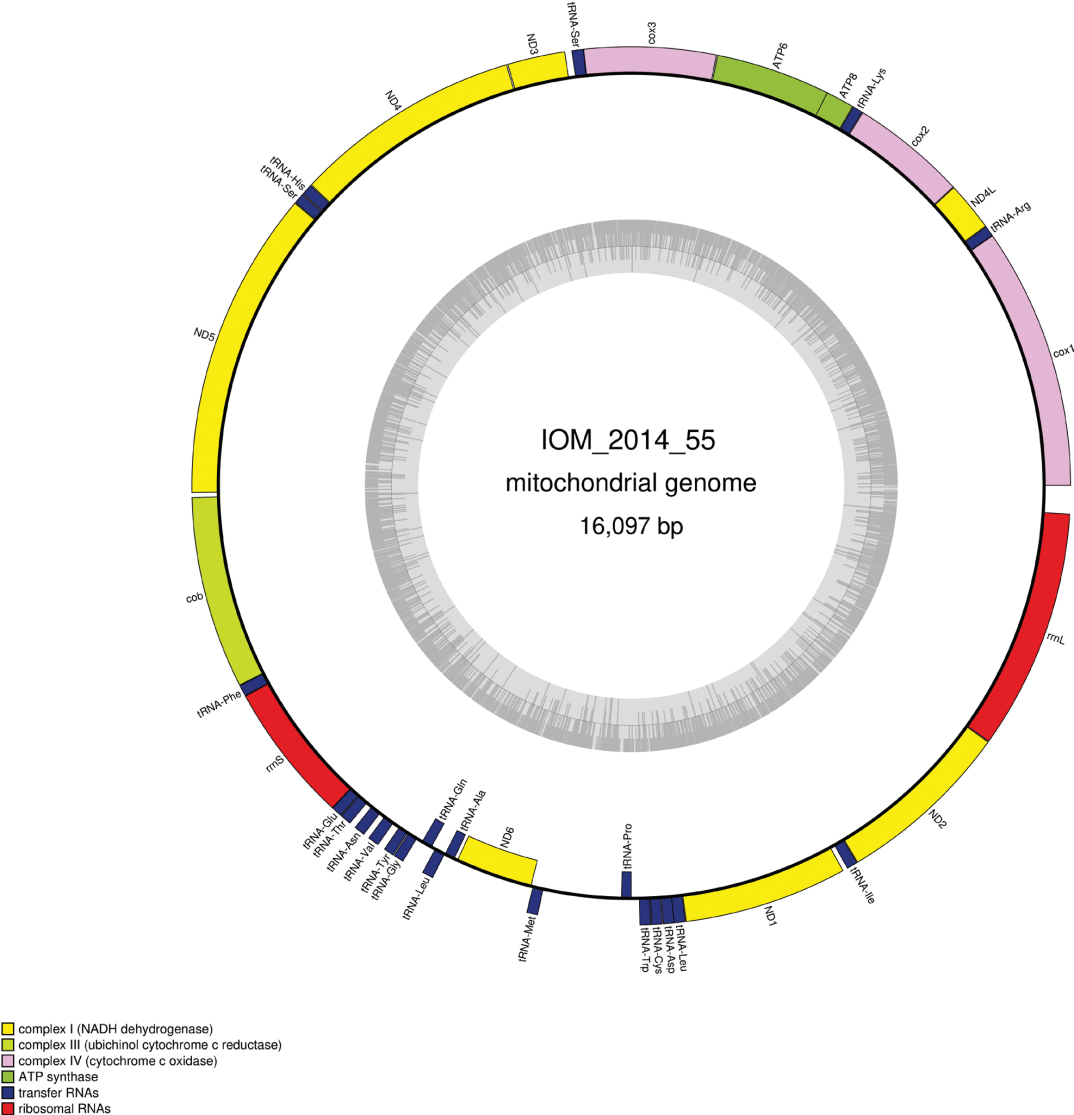
Map of the mitochondrial genome of specimen IOM_2014_55, with the type of genes indicated by colour boxes and the GC content indicated by the grey circle.

The mitogenome is complete with redundant endings (GenBank: PP977507). It is 16,097 bp long and encodes 13 protein coding genes, two rRNAs and 22 tRNAs (Fig. 17, Table 9). The nucleotide composition is A (35.23%), T (33.57%), C (17.98%) and G (13.22%). The best *cox1* megablast result is *Isostichopusbadionotus* Selenka, 1867 (GenBank: MZ188901) (Drake et al. 2021), with E-value 0.0, identity 79.58% for a length of 16,318 bp (the sequence represents a complete mitochondrial genome). When aligning the *cox1* gene with sequences of various Holothuroidea from the CCZ as studied by Bribiesca-Contreras et al. (2022) and performing an ML phylogeny on them (model of evolution GTR+I+G4), the tree reveals that specimen IOM_2014_55 is sister to a clade containing *Oneirophanta* sp. CCZ 100 voucher CCZ_100 (ON400706) and Oneirophantacf.mutabilis GBC-2022 voucher CCZ_193 (ON400724) with a 89% support at the node (tree not shown).

**Table 9.** Characteristics of the genes encoded by the mitogenome the unidentified Holothuroidea IOM_2014_55. T(AA) in the stop codon column indicates a premature termination with the addition of 3’ A residues to the mRNA.

Gene	Strand	Location	Size (bp)	Start codon	Stop codon	Anticodon
*cox1*	+	1–1554	1554	ATG	TAA	
*tRNA-Arg*	+	1553–1618	66			TCG
*ND4L*	+	1619–1915	297	ATG	TAA	
*cox2*	+	1916–2604	689	ATG	TA(A)	
*tRNA-Lys*	+	2605–2668	64			CTT
*ATP8*	+	2669–2842	174	ATG	TAA	
*ATP6*	+	2830–3519	690	ATG	TAA	
*cox3*	+	3522–4304	783	ATG	TAA	
*tRNA-Ser*	+	4303–4373	71			TGA
*ND3*	+	4412–4756	345	ATG	TAA	
*ND4*	+	4760–6116	1357	ATG	T(AA)	
*tRNA-His*	+	6118–6186	69			GTG
*tRNA-Ser*	+	6188–6254	67			GCT
*ND5*	+	6255–8090	1836	ATG	TAA	
*cob*	+	8155–9257	1103	ATG	TAA	
*tRNA-Phe*	+	9257–9327	71			GAA
*rrnS*	+	9326–10159	834			
*tRNA-Glu*	+	10158–10224	67			TTC
*tRNA-Thr*	+	10225–10294	70			TGT
*tRNA-Asn*	+	10330–10398	69			GTT
*tRNA-Val*	+	10431–10500	70			TAC
*tRNA-Tyr*	+	10543–10608	66			GTA
*tRNA-Gly*	+	10611–10675	65			TCC
*tRNA-Gln*	-	10711–10780	70			TTG
*tRNA-Leu*	+	10804–10873	70			TAG
*tRNA-Ala*	-	10873–10939	67			TGC
*ND6*	-	10958–11446	489	ATG	TAG	
*tRNA-Met*	+	11456–11524	69			CAT
*tRNA-Pro*	-	12010–12075	66			TGG
*tRNA-Trp*	+	12121–12189	69			TCA
*tRNA-Cys*	+	12190–12256	67			GCA
*tRNA-Asp*	+	12258–12325	68			GTC
*tRNA-Leu*	+	12321–12391	71			TAA
*ND1*	+	12392–13363	972	ATG	TAA	
*tRNA-Ile*	+	13389–13456	68			GAT
*ND2*	+	13457–14503	1047	ATG	TAA	
*rrnL*	+	14504–15933	1430			

### ﻿Specimen IOM_2014_57: unidentified Porifera

Fig. 18.

**Figure 18.**
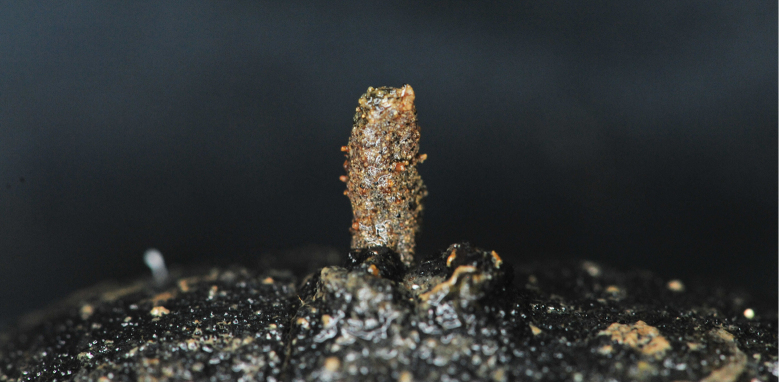
Specimen IOM_2014_57 on a polymetallic nodule immediately after sampling (unscaled).

Station ID: 3521Tr_1.

Biosample: SAMN42180859.

#### ﻿Cluster of nuclear rRNA genes

The cluster is complete with a total length of 5,804 bp (GenBank: PP968769). The best *18S* megablast result is again *P.pachymastia* voucher UCMPWC932 (GenBank: EF654528), E-value 0.0, identity 99.76% for a length of 1,660 bp. Megablast queries of the D1/D2 region returned a 100% identity with *Tentorium* sp. voucher NHM1404 (GenBank: PP848927) and *Tentorium* sp. voucher NHM1619 (GenBank: PP848930).

#### ﻿Mitochondrial genome

**Figure 19.**
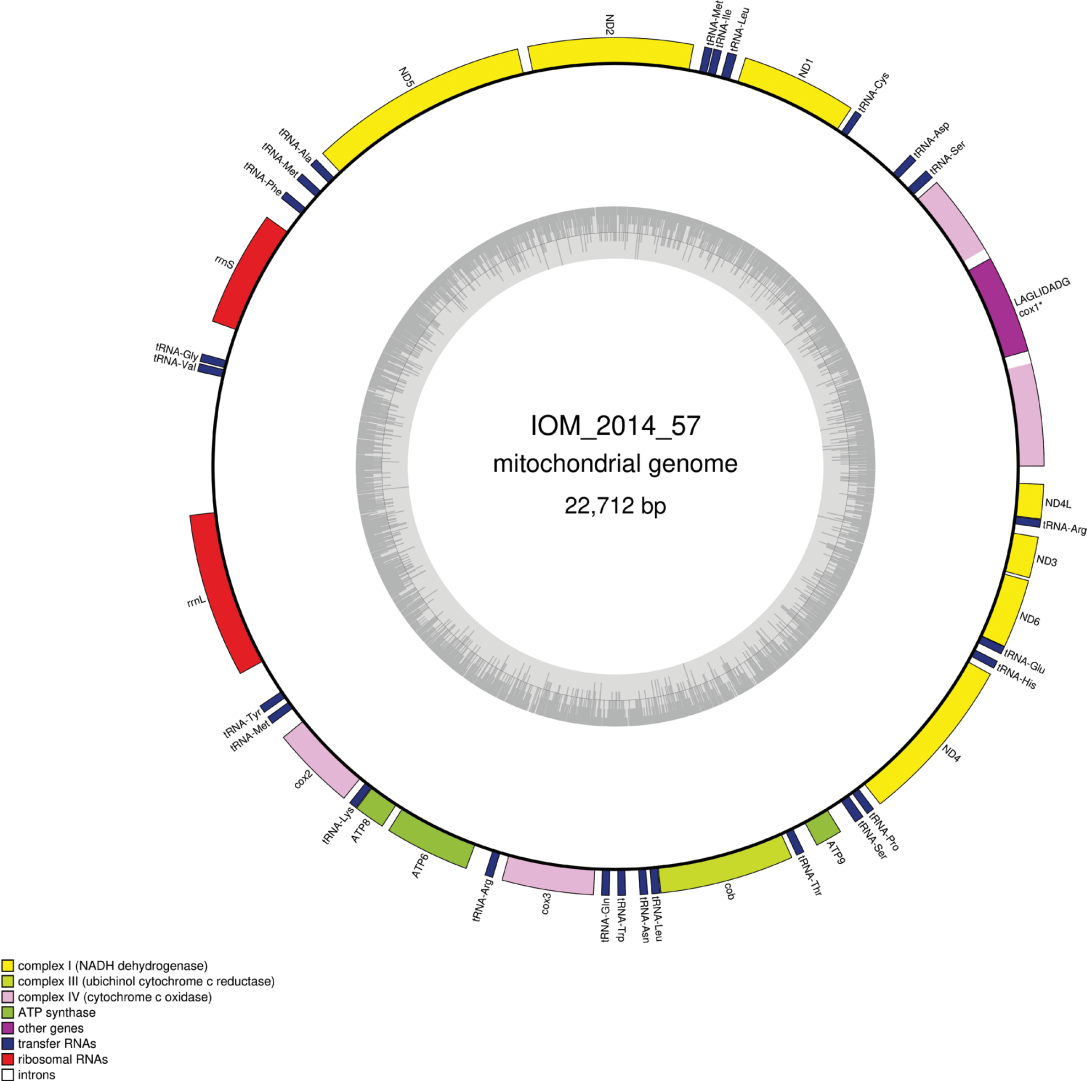
Map of the mitochondrial genome of specimen IOM_2014_57, with the type of genes indicated by colour boxes and the GC content indicated by the grey circle. Introns appear in white.

The mitogenome is complete with redundant endings (GenBank: PP971518). It is 22,712 bp long and encodes 14 protein coding genes, two rRNAs and 25 tRNAs, all on the same strand (Fig. 19, Table 10). The nucleotide composition is A (31.35%), T (36.60%), C (12.30%) and G (19.75%). There is a group I intron in the *cox1* gene that contains a 282 amino-acid long ORF encoding a putative LAGLIDADG endonuclease. The best megablast result for the CDS of the *cox1* gene is *P.littoralis* (GenBank: KJ129611) with E-value 0.0, identity 94.81% for a length of 21,719 bp (representing a complete mitogenome).

**Table 10.** Characteristics of the genes encoded by the mitogenome the unidentified Porifera IOM_2014_57. The size of the intron-containing genes is indicated with and without the introns.

Gene	Strand	Location	Size (bp)	Start codon	Stop codon	Anticodon
*cox1*	+	1–2620 (1 intron)	2620 (full) 1563 (CDS)	ATG	TAG	
*tRNA-Ser*	+	2669–2752	84			TGA
*tRNA-Asp*	+	2868–2939	72			GTC
*tRNA-Cys*	+	3467–3532	66			GCA
*ND1*	+	3571–4566	996	ATG	TAG	
*tRNA-Leu*	+	4640–4723	84			TAA
*tRNA-Ile*	+	4772–4844	73			GAT
*tRNA-Met*	+	4853–4923	71			CAT
*ND2*	+	5013–6428	1416	ATG	TAA	
*ND5*	+	6509–8401	1893	ATG	TAG	
*tRNA-Ala*	+	8470–8542	73			TGC
*tRNA-Met*	+	8640–8711	72			CAT
*tRNA-Phe*	+	8843–8915	73			GAA
*rrnS*	+	9111–10109	999			
*tRNA-Gly*	+	10393–10464	72			TCC
*tRNA-Val*	+	10478–10550	73			TAC
*rrnL*	+	11766–13180	1415			
*tRNA-Tyr*	+	13494–13564	71			GTA
*tRNA-Met*	+	13609–13680	72			CAT
*cox2*	+	13824–14555	732	ATG	TAA	
*tRNA-Lys*	+	14612–14684	73			TTT
*ATP8*	+	14686–14955	270	ATG	TAA	
*ATP6*	+	15015–15749	735	ATG	TAA	
*tRNA-Arg*	+	15912–15985	74			TCT
*cox3*	+	16062–16850	789	ATG	TAG	
*tRNA-Gln*	+	16913–16984	72			TTG
*tRNA-Trp*	+	17051–17121	72			TCA
*tRNA-Asn*	+	17238–17308	71			GTT
*tRNA-Leu*	+	17339–17413	75			TAG
*cob*	+	17415–18569	1155	ATG	TAA	
*tRNA-Thr*	+	18609–18682	74			TGT
*ATP9*	+	18799–19035	237	ATG	TAA	
*tRNA-Ser*	+	19178–19262	85			GCT
*tRNA-Pro*	+	19300–19372	73			TGG
*ND4*	+	19436–20887	1452	ATG	TAA	
*tRNA-His*	+	20939–21011	73			GTG
*tRNA-Glu*	+	21075–21146	72			TTC
*ND6*	+	21144–21740	597	ATG	TAA	
*ND3*	+	21760–22116	357	ATG	TAA	
*tRNA-Arg*	+	22194–22264	71			TCG
*ND4L*	+	22265–22564	300	ATG	TAG	

### ﻿Specimen IOM_2014_62: unidentified Brachiopoda

Fig. 20.

**Figure 20.**
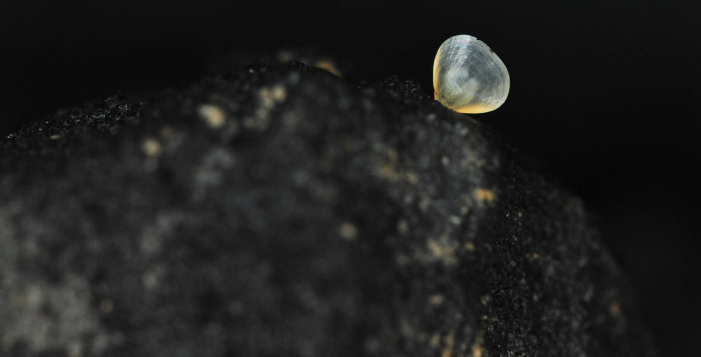
Specimen IOM_2014_62 on a polymetallic nodule immediately after sampling (unscaled).

Station ID: 3545.

Biosample: SAMN42180860.

#### ﻿Cluster of nuclear rRNA genes

It was impossible to obtain the complete cluster of rRNA, or the complete *18S*. Only a 809 bp fragment could be derived from the assembly (GenBank: PP971154). The best *18S* megablast result is *Neorhynchia* sp. D1090 (GenBank: AF025937) (Cohen et al. 1998), E-value 0.0, identity 99.88% for a length of 1,769 bp.

#### ﻿Mitochondrial genome

**Figure 21.**
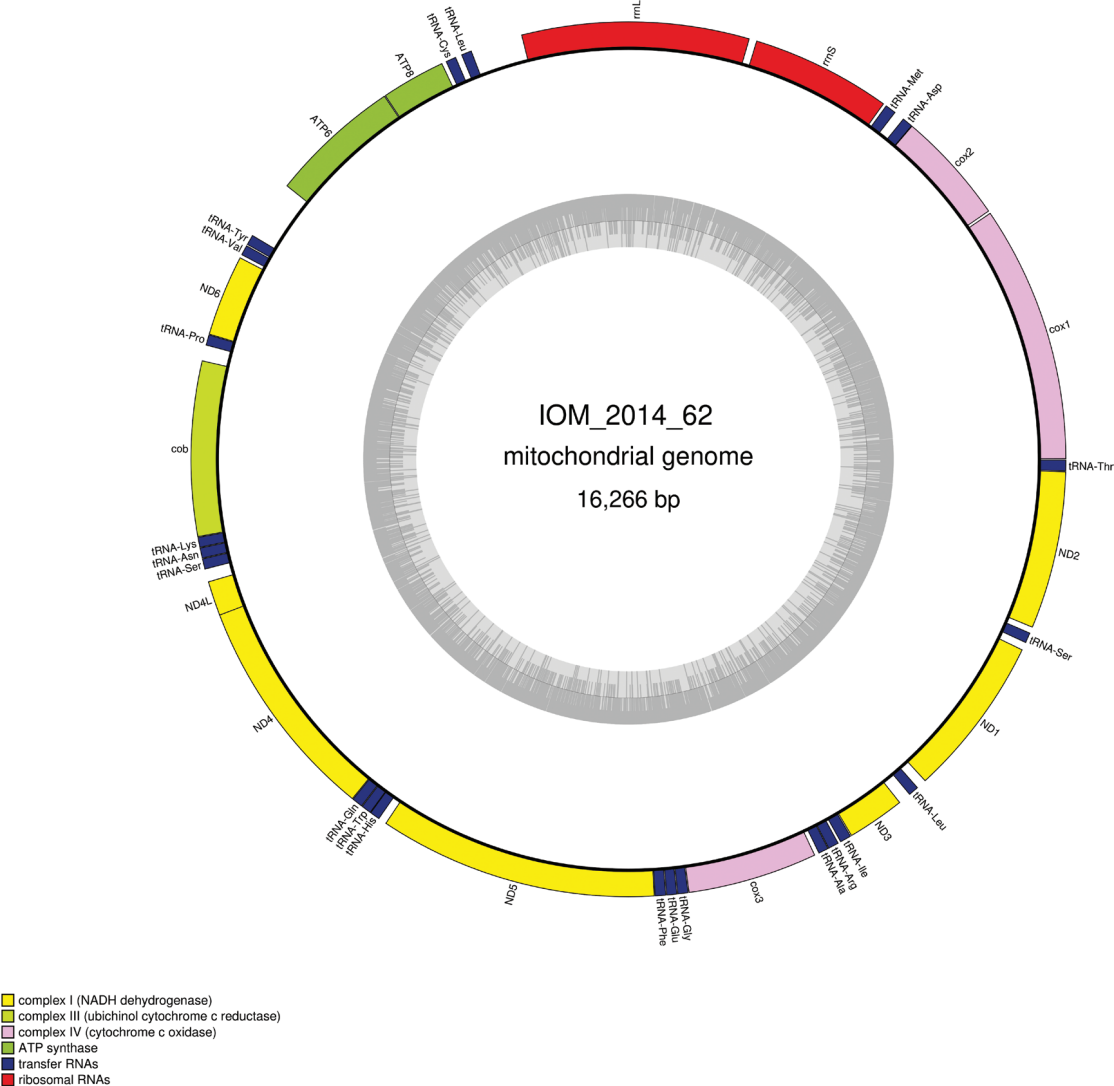
Map of the mitochondrial genome of specimen IOM_2014_62, with the type of genes indicated by colour boxes and the GC content indicated by the grey circle.

The mitogenome is complete with redundant endings (GenBank: PP977509). It is 16,266 bp long and codes for 13 protein coding genes, two rRNAs and 22 tRNAs, all encoded on the same strand (Fig. 21, Table 11). The nucleotide composition is A (28.05%), T (26.52%), C (30.14%) and G (15.29%). The best *cox1* megablast result is *Hemithiris* sp. Hem1 (GenBank: AB026517) (Saito et al. 2000) with E-value 0.0, identity 81.71% for a length of 1,218 bp.

**Table 11.** Characteristics of the genes encoded by the mitogenome the unidentified Holothuroidea IOM_2014_62. T(AA) in the stop codon column indicates a premature termination with the addition of 3’ A residues to the mRNA.

Gene	Strand	Location	Size (bp)	Start codon	Stop codon	Anticodon
*cox1*	+	1–1548	1548	ATG	TAA	
*cox2*	+	1560–2243	684	ATG	TAA	
*tRNA-Asp*	+	2242–2309	68			GTC
*tRNA-Met*	+	2368–2437	70			CAT
*rrnS*	+	2453–3298	846			
*rrnL*	+	3340–4707	1368			
*tRNA-Leu*	+	5016–5081	66			TAG
*tRNA-Cys*	+	5116–5181	66			GCA
*ATP8*	+	5205–5594	390	ATG	TAA	
*ATP6*	+	5596–6387	792	ATC	TAG	
*tRNA-Tyr*	+	6741–6806	66			GTA
*tRNA-Val*	+	6811–6875	65			TAC
*ND6*	+	6892–7384	493	ATG	T(AA)	
*tRNA-Pro*	+	7385–7451	67			TGG
*cob*	+	7543–8595	1053	ATG	TAA	
*tRNA-Lys*	+	8596–8659	64			TTT
*tRNA-Asn*	+	8660–8725	66			GTT
*tRNA-Ser*	+	8726–8792	67			TGA
*ND4L*	+	8866–9078	213	ATG	TAA	
*ND4*	+	9072–10434	1363	ATG	T(AA)	
*tRNA-Gln*	+	10435–10503	69			TTG
*tRNA-Trp*	+	10504–10567	64			TCA
*tRNA-His*	+	10567–10630	64			GTG
*ND5*	+	10676–12355	1680	ATT	TAA	
*tRNA-Phe*	+	12356–12423	68			GAA
*tRNA-Glu*	+	12425–12489	65			TTC
*tRNA-Gly*	+	12491–12556	66			TCC
*cox3*	+	12558–13340	783	ATG	TAA	
*tRNA-Ala*	+	13364–13428	65			TGC
*tRNA-Arg*	+	13429–13496	68			TCG
*tRNA-Ile*	+	13509–13580	72			GAT
*ND3*	+	13583–13933	351	GTG	TAG	
*tRNA-Leu*	+	14005–14070	66			TAA
*ND1*	+	14127–15101	975	ATA	TAA	
*tRNA-Ser*	+	15145–15210	66			TCT
*ND2*	+	15244–16194	951	ATC	TAA	
*tRNA-Thr*	+	16196–16263	68			TGT

## ﻿Discussion

It is assessed that there is a substantial gap between the CCZ biodiversity and the described metazoan species. Many species remain not only to be described, but also to be discovered (Amon et al. 2016; Christodoulou et al. 2019; Rabone et. al 2023). The gap is being slowly filled in, thanks to the increasing number of sampling efforts undertaken by scientist over the past years (including scientists working with ISA contractors), but at the same time new areas of knowledge gaps are being identified. To a certain extent, this paradox is reflected in the increasing amount of environmental data that ISA contractors are required to collect. Comparison of the LTC recommendations from previous years with the most recent ones clearly underlines the gaps in our knowledge (ISBA/25/LTC/6/Rev.3). This includes but is not limited to the application of genetic studies in assessing benthic biodiversity and population connectivity of organisms.

Although molecular studies have been making rapid progress over past years, advancing our knowledge on benthic metazoans, there are still phyla that have received limited attention from a genomic point of view. This is the case for Brachiopoda for example, which has been scarcely documented so far. Among the more than 400 known non-fossil species of Brachiopoda, fewer than ten have had their mitogenomes sequenced, with the majority of the sequences belonging to inarticulate brachiopods (Karagozlu et al. 2021; Niaison et al. 2021; Breton 2024). Only four species (and three genera) of articulate taxa, to which specimen IOM_2014_62 is likely to belong, have had their mitogenomes sequenced and published (Stechmann and Schlegel 1999; Helfenbein et al. 2001; Karagozlu et al. 2017; Noguchi et al. 2000). The percentage of identity of the *cox1* gene between specimen IOM_2014_62 and the four other species ranges between 63.27% and 72.31%. There is a practical implication of such differences: DNA primers designed using previously published reference mitogenomes may not anneal correctly on the DNA of a specimen such as IOM_2014_62, and possibly other Brachiopoda from the CCZ. Documenting these taxa with more mitogenome data could help solve this problem, with the subsequent possibility to design more efficient primers.

Among our specimens, half of the Porifera and Bryozoa have introns in their mitogenomes, in each case in the *cox1* gene. Although rare and nearly absent in other taxa groups, this is not the first time that introns have been found within the mitogenomes of these two phyla (Rot et al. 2006; Jenkins et al. 2022). In the case of Porifera, intron content within a single species has been proven to vary across populations (Cranston et al. 2021). Not only are these findings interesting from the evolutionary genomics point of view but, due to the unpredictable presence of introns, they also challenge the use of the *cox1* gene for routine molecular barcoding of the CCZBryozoa and Porifera (Neal et al. 2022, 2023). Owing to the presence of introns, amplification of this gene by PCR might fail, or at least will require adoption of a protocol for longer elongation time and possibly the use of the Taq polymerase suitable for a long PCR.

A solution to such issues might be our genome-skimming approach. However, this approach has its limitations, one of them being that obtaining the required amount of DNA could result in the destruction of the smallest samples. There could thus be a risk of not leaving a correct specimen voucher behind, which is not in line with the ISA recommendations that advocate for reverse taxonomy followed by curation of voucher specimens and molecular samples in order to maintain the link between morphology-based and molecular-based identifications (ISBA/25/LTC/6/Rev.3 2023). Otherwise, such approach might require a preliminary treatment such as whole genome amplification. Regardless of the above limitation, when specimens qualify in terms of biomass, or are expendable because of their limited further use (which was the case with some of our own material), our approach could still be applied.

Among the ten specimens in this study, five of the sequences obtained matched the sequences stored in GenBank. Sequencing confirmed that specimen IOM_2014_38 is *S.daleus* and that specimens IOM_2014_54 and IOM_2014_58 were *O.glabrum*. We regard as especially promising the results obtained on *O.glabrum*, for further studies in the emerging field of population genetics and connectivity in the CCZ (Taboada et al. 2018; Riehl and De Smet 2020). With 12 SNPs out of 1602 bp, the *cox1* gene would be a useful population marker for this species, as already suggested by the works of Christodoulou et al. (2020). It could be noted that none of the sequences obtained by Christodoulou et al. (2020) were identical to the *cox1* gene of IOM_2014_54 and IOM_2014_58, which suggests a large polymorphism of this gene among this species.

The two other specimens which matched to some degree with GenBank references were identified as Demospongiae (IOM_2014_13) and Polychaeta (IOM_2014_17). Both were far more degraded, especially IOM_2014_17, which was torn into two pieces of ~ 1.5 cm each. Neither of the specimens was suitable for taxonomy, neither preliminary nor reverse. Megablast queries returned a 99.28% identity of IOM_2014_17 with Nicomachecf.benthaliana NHM_058, an organism that has been previously found in the licence areas UK-1A and UK-1B (UK Seabed Resources Ltd.), BGR (Federal Institute for Geosciences and Natural Resources of the Federal republic of Germany) and OMS (Ocean Mineral Singapore PTE Ltd.), all of which are located to the East of IOM claim area (Stewart et al. 2023). It is also worth reminding that queries of partial *18S*, *28S*, and *cox1* returned a complete identity between IOM_2014_13 and *Spinularia* sp. voucher RC1570 which was also sampled in the Eastern part of the CCZ, as for the aforementioned Polychaeta.

The results of genome comparison obtained for specimen of Demospongiae IOM_2014_13 are more intriguing. The 100% identity with the partial *cox1* gene of *S.sarsii* poses some problems. As far as we know, this species has never been reported in the CCZ. It is mostly found in the Atlantic Ocean, and a few locations have also been reported for the South-West Pacific (de Voogd et al. 2024). If the presence of *S.sarsii* is confirmed in the CCZ, it will raise questions about its global distribution. However, no further assessment should be done for this species based on our sequencing results, as we are faced with two taxonomic issues. First, it was impossible to perform a correct morphology-based identification of a partial and degraded specimen. Second, it should be noted that the 100% identity of the *cox1* gene was returned for a 658 bp fragment deposited in GenBank, which is less than half the length of the complete *cox1* gene of Demospongiae IOM_2014_13, leaving room for informative polymorphisms outside this 658 bp fragment. Moreover, the differences in length between queries and the sequences registered in GenBank may lead to further difficulties. When results are sorted based on their ‘Max Score’ or ‘Total Score’, different lengths and the impact they have on the ‘Query Cover’ parameter affect the result returned by such query. If the reference sequence deposited in GenBank is considerably shorter than the query, it may lead to the exclusion of the reference from the sequences producing significant alignment, whose number is limited to a maximum of one hundred. This in fact could serve as an argument in favour of our genome-skimming approach: submitting complete mitogenomes to GenBank means that querying a partial gene belonging to an identical species from the CCZ will return complete sequences as top results. With this in mind, we hope that the results presented here could be used as references in future studies on phylogeny, distribution of species and possibly population genetics of benthic organisms inhabiting the CCZ. We also hope that further investigations by other teams would lead to a more formal identification or description of the unidentified taxa here studied, and that such studies will benefit from the genomic results here presented.

## ﻿Additional information

### Author ORCIDs

Romain Gastineau https://orcid.org/0000-0001-8661-5118

Kamila Mianowicz https://orcid.org/0000-0002-7755-3258

Przemysław Dąbek https://orcid.org/0000-0002-3736-3011

Christian Otis https://orcid.org/0000-0001-9680-5863

Tomasz Abramowski https://orcid.org/0000-0002-9029-406X

### Data availability

All of the data that support the findings of this study are available in the main text.
